# Novel 3D Echocardiographic Technique for Mitral Calcium Mapping

**DOI:** 10.3390/jcm12041470

**Published:** 2023-02-12

**Authors:** Francesca Romana Prandi, Francesco Romeo, Francesco Barillà, Samin Sharma, Annapoorna Kini, Stamatios Lerakis

**Affiliations:** 1 Department of Cardiology, Mount Sinai Hospital, Icahn School of Medicine at Mount Sinai, New York, NY 10029, USA; 2 Division of Cardiology, Department of Systems Medicine, Tor Vergata University, 00133 Rome, Italy; 3 Faculty of Medicine, Unicamillus-Saint Camillus International University of Health and Medical Sciences, 00131 Rome, Italy

**Keywords:** mitral annular calcification, 3D echocardiography, calcific mitral valve disease, structural heart disease imaging, mitral valve intervention

## Abstract

Mitral annular calcification (MAC) is a common chronic degenerative process of the mitral valve fibrous support ring. MAC increases the risk of mitral valve dysfunction, all-cause and cardiovascular mortality, and worse outcomes in cardiac interventions. Echocardiography represents the first imaging modality for MAC assessment, but it has low specificity compared to cardiac CT in terms of distinguishing between calcium and dense collagen. Novel three-dimensional transesophageal maximal intensity projection (MIP) mapping allows for the real-time MAC distribution and depth visualization of the cardiac anatomy and represents a useful and promising tool for pre-procedural assessment and intra-procedural guidance of cardiac interventions.

## 1. Introduction

Mitral annular calcification (MAC) is a chronic degenerative process that results from the progressive calcification of the mitral annulus. The mitral annulus is a saddle-shaped complex structure that separates the left atrium from the left ventricle; it is in close continuity with the aortic root and aorto-mitral curtain anteriorly and it is discontinuous and periodically interrupted by fat tissue posteriorly. MAC is a common finding in cardiovascular imaging studies, with an estimated prevalence of 9–15% in the general population [1,2,3] and of 42% in elderly patients [4].

The etiopathogenesis of MAC is multifactorial, involving abnormal calcium and phosphorus metabolism, increased hemodynamic stress on the mitral valve, atherosclerotic processes [5], and inflammation [6]. Risk factors for MAC include aging, female sex, chronic kidney disease, diabetes mellitus, increased body mass index, prior chest irradiation, metabolic disorders, osteoporosis, and conditions that predispose to left ventricular hypertrophy and increased mitral valve stress, such as hypertension, aortic stenosis, hypertrophic cardiomyopathy, and mitral valve prolapse. MAC can also be present alongside congenital metabolic disorders such as Marfan syndrome and Hurler syndrome [1,2,3,4,5,6,7]. In Marfan syndrome, the mitral annular calcification process might be secondary to an increased mitral valve stress associated with mitral valve prolapse or due to a connective tissue intrinsic abnormality, while in Hurler syndrome it may be caused by fibroblasts abnormalities and accelerated collagen degeneration [7].

Calcific mitral valve disease is typically confined to the mitral annulus (more commonly on the posterior portion of the annulus [8], typically extending from the lateral to the medial fibrous trigones, but in 1.5% of patients it can be circumferential [5]) and the bases of the leaflets. Over time, it may further expand into the leaflets, resulting in geometric distortion and impaired mobility [5]. In some patients, it can also extend to the ventricular myocardium, papillary muscles [8], aorto-mitral curtain, aortic valve [6], and left atrial wall (“porcelain left atrium”) [9]. Occasionally, calcium deposits are mobile and can embolize [10].

Rarely, extensive MAC may result in mitral stenosis (MS), which has been termed “degenerative MS” to differentiate it from the rheumatic etiology. Indeed, unlike in rheumatic mitral valve involvement, in MAC, the leaflets’ commissures are usually spared. Occasionally, MAC may lead to mitral regurgitation (MR), left ventricular outflow tract (LVOT) obstruction, and endocarditis [11]. MAC was found to be independently associated with a higher risk of all-cause mortality, cardiovascular mortality [12], coronary artery disease [13], incident congestive heart failure [14], stroke [15], conduction system abnormalities [16], and atrial fibrillation [17]. For each 1 mm increase in MAC, there is a 10% increased risk of incident cardiovascular disease, cardiovascular death, and all-cause death [12].

MAC also influences the outcomes of cardiac surgery and transcatheter valve interventions. The prognosis is poor for the overall higher-risk profile of these patients and for the technical anatomical challenges related to the presence of annular calcification, entailing increased morbidity and mortality rates. MAC influences mitral valve repair feasibility, and it is associated with increased intraoperative conversion from repair to replacement and worse outcomes of mitral valve surgery [18]. Surgical mitral valve replacement (SMVR) currently represents the preferred management option for patients with severe mitral valve dysfunction that are symptomatic and present low/intermediate surgical risk, even in the presence of severe MAC [19]. Patients with extensive calcification of the mitral annulus who require mitral valve replacement or repair surgery have an increased risk of atrioventricular junction and LV free wall rupture [20], circumflex artery injury [7], and embolization during MAC debridement. In mitral valve replacement, MAC severely interferes with the suture anchoring of the prosthetic valve, increasing the risk of paravalvular leak [21]. For these reasons, patients with severe MAC are often considered to have too high of a risk to undergo surgery. On the other side, subjects with significant mitral valve dysfunction due to any degree of MAC have poor survival if left untreated, with a higher all-cause mortality and composite outcome of mortality or heart failure hospitalization at 3-year follow-up in the patients that did not undergo mitral intervention [21]. Transcatheter mitral valve replacement (TMRV) is emerging as an alternative treatment for patients with severe MAC and mitral valve dysfunction who are symptomatic and have high or prohibitive surgical risk and appropriate anatomy, although it presents specific challenges including paravalvular leaks and LVOT obstruction [22]. Valve embolization represents another intraprocedural risk of the transcatheter approach, and it is due mainly to the use of an undersized device or to insufficient MAC necessary for ensuring adequate valve anchoring [22]. Moderate or severe MAC were not associated with decreased procedural success, durability of repair, or left ventricular remodeling in the transcatheter edge-to-edge repair (TEER) of MR with the MitraClip system [23], but TEER for calcific MR is often limited by pre-existing MS or leaflet calcification that makes leaflet grasping difficult. The proposed anatomic criteria for MitraClip candidacy have excluded patients with a mitral valve area <4 cm^2^ and severe MAC [24], and an elevated pre-procedural mean diastolic gradient and the presence of MAC were demonstrated to be independent predictors of elevated post-procedural mean diastolic gradient [25]. Half of all patients undergoing transcatheter aortic valve replacement (TAVR) have MAC [26]. Severe MAC is a strong predictor of all-cause and cardiovascular mortality after TAVR and it represents an independent strong predictor of the success of new, permanent pacemaker implantation after TAVR, so its inclusion in future TAVR risk stratification models should be considered [26]. The percutaneous and surgical treatment options for this challenging patient group are rapidly evolving, and novel diagnostic technologies are needed to improve care for this population [5].

## 2. Mitral Annular Calcification Imaging

Conventionally, echocardiography represents the first-line imaging modality for the assessment of calcific mitral valve disease [5], although, in comparison with cardiac computed tomography (CT), it is less suited for MAC detection because of its relatively low specificity with respect to distinguishing between calcium and dense collagen [1].

MAC is usually visualized on echocardiography as an echo-dense structure with an irregular appearance and associated acoustic shadowing [7]. Transthoracic two-dimensional (2D) echocardiography (TTE) allows experts to assess the qualitative extent of annular calcification (focal versus circumferential), which is best achieved via short-axis views of the mitral valve. In addition, TTE evaluation allows for the detection of associated extra-annular involvement, the presence of mobile elements, and mitral valve dysfunction [27]. TEE is useful for the further assessment of the mechanisms and severity of associated mitral valve dysfunction. Currently, there is not a universally accepted classification system of MAC severity. MAC severity is typically graded echocardiographically as mild (<1/3 annular circumference involved), moderate, or severe (>1/2 annular involvement) [4]. MAC that protrudes into the LV inlet [4] or that is more than 4 mm in thickness (measured in the anteroposterior direction in the short-axis view) [28] is also graded as severe.

Cardiac CT is a well-established tool for coronary calcium detection, and it has shown higher accuracy than other imaging modalities with respect to the detection of cardiac calcifications due to the high spatial resolution of CT and high X-ray attenuation of calcification [28]. Cardiac CT currently represents the best imaging technique with which to evaluate MAC’s location, distribution, quantification (calcium score via the Agatston method), and extent (MAC calcification angle). The circumferential extent of calcifications is best viewed using multiplanar reformatting to create a short-axis view of the mitral valve annulus [5]. Cardiac CT also identifies extra-annular calcification involvement (LVOT extension and infiltration into the myocardium) and it is especially useful for the detection of the caseous calcification of the mitral annulus [29]. Therefore, cardiac CT is becoming an integral tool in the planning of mitral valve procedures [7]. The routine use of cardiac CT in the diagnostic work-up of patients evaluated for cardiac surgery or transcatheter valve interventions may also help determine the true incidence and clinical implications of MAC [7]. A limitation of CT is that it does not allow for the functional evaluation of the mitral valve [5].

## 3. Novel 3D Echocardiographic Technique for MAC

Over the last two decades, the number of real-time, three-dimensional (3D) echocardiography applications in clinical cardiology has grown exponentially, showing promising future directions, including with respect to 3D printing and virtual reality [30]. An important advantage of three-dimensionality is its facilitation of the measurement of an object in arbitrary orientations without geometric assumptions.

En face atrial and ventricular views of the mitral valve via 3D imaging provide the best topographical information on mitral valve diseases, demonstrate significant alterations in annular and leaflets mechanics, and allow for the direct planimetry of the MV area [5] as well as the detection and improved quantification of associated MS and MR.

In 3D-echocardiography, high gain causes images to appear more two-dimensional, while low gain reveals deeper tissue; high compression makes an image transparent, and low compression makes an image more solid [31].

Volume-rendering techniques project 3D data sets on a two-dimensional display. The standard mode of the two-dimensional display is a colorized depth map, which cannot be used to accurately distinguish MAC from dense collagen; a translumination map presents the same limitation. By changing it to a maximal intensity projection (MIP) map, it is possible to visualize the calcium distribution and depth without losing sight of the surrounding tissues and their anatomical relations [32].

An MIP map is very simple: each rendered pixel is the highest intensity voxel along the raycast line, such as a simulated X-ray. The intensity of each voxel along the raycast line is a measure of the scattering properties at that location. This map is actually the product of the scattering properties and the isonification energy, although changes in isonification energy are compensated with time gain compensation. The scattering properties are related to the density but are not a direct measure of the density. Calcium has scattering properties that are reflective of a hard substance that is continuous in comparison to the wavelength of the ultrasound [30]. Therefore, with an MIP map, calcium appears as a continuous, bright target forming irregular and lumpy areas compared with the surrounding tissues without calcifications, while also maintaining anatomical relations. This allows for the facile identification of the presence, distribution, and extent of MAC.

## 4. Maximal Intensity Projection Map Applications

An MIP map enables the real-time and three-dimensional evaluation of the calcium distribution and depth on the mitral annulus. Therefore, it represents a promising tool that can be very useful in clinical practice, since information on the presence, distribution, and extent of calcium are critical for the planning of cardiac interventions and mitral surgery.

**Figure 1:**
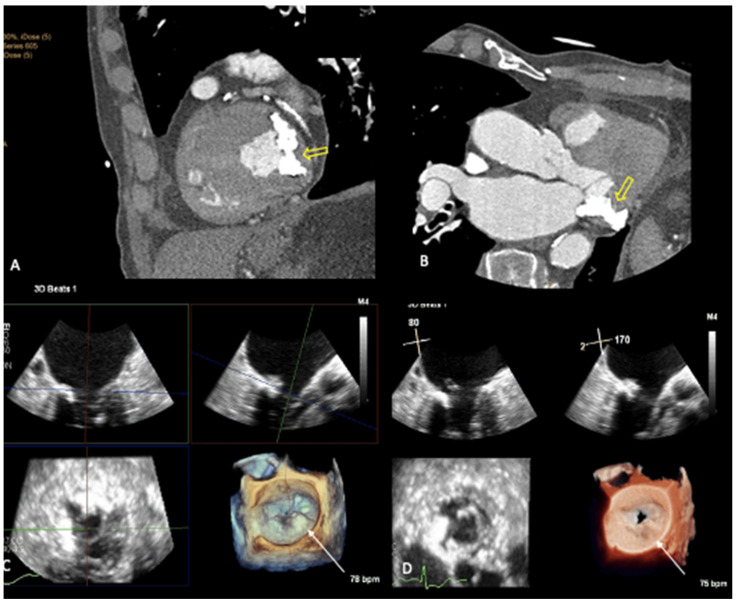
Cardiac computed tomography scan from a patient with severe circumferential mitral annular calcification (MAC) with caseous necrosis extending posteriorly beyond the annulus (**A**,**B**, yellow arrows). Transesophageal echocardiogram (TEE) from the same patient, documenting MAC in two-dimensions (2D) and mitral annular irregularity in three-dimensional (3D) volume-rendering colorized-depth map (**C**, white arrow) and transillumination map (**D**, white arrow).

**Figure 2:**
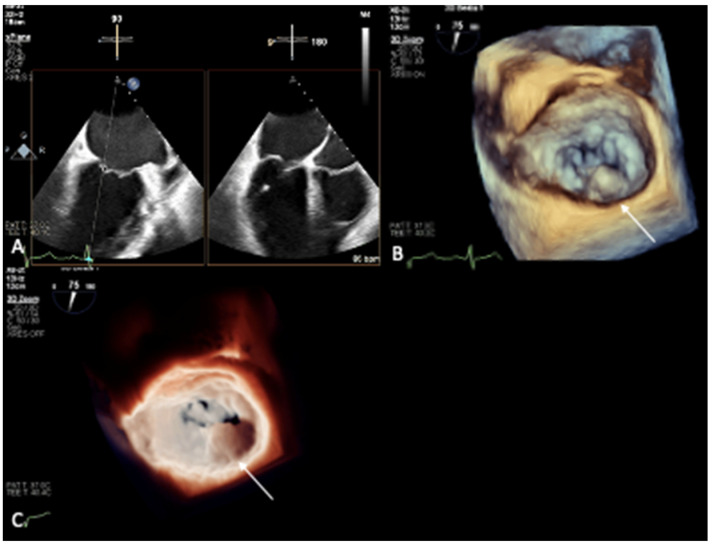
TEE from the control patient, documenting no MAC in 2D (**A**) and mitral annular irregularity in 3D volume-rendering colorized-depth map (**B**, white arrow) and transillumination map (**C**, white arrow), demonstrating the low specificity of these maps with respect to distinguishing between calcium and dense collagen.

**Figure 3:**
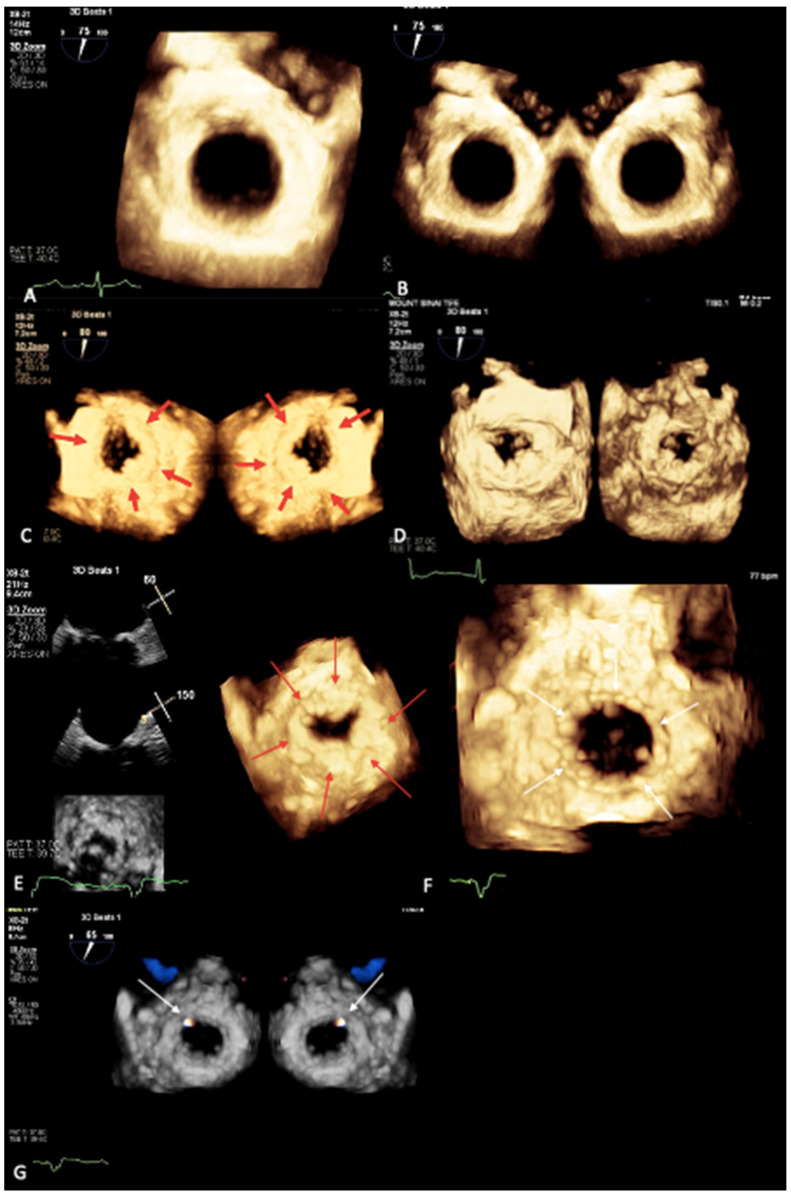
Novel 3D TEE maximal intensity projection map (MIP) allows for the exclusion of the presence of MAC in the control patient (surgeon’s view, **A**; atrial and ventricular view, **B**), where the mitral annulus appears impressively smooth, unlike the MIP map in the patient with MAC (atrial and ventricular view, **C**, where the red arrows indicate MAC, and **D)**, where the mitral annulus appears irregular and lumpy). During transcatheter valve-in-MAC procedure, MIP tool was able to visualize in real time the circumferential distribution and extent of MAC (**E**, red arrows indicate MAC), thus guiding positioning and deployment of the valve, and post-deployment MIP map clearly delineated the circular shape of the valve frame opposed to the surrounding calcium, without signs of significant gaps, allowing for the exclusion of significant paravalvular leak (PVL) and the need for post-dilation (**F**, white arrows indicate the valve frame). Color-Doppler on MIP confirmed only trivial PVL at 1 o’ clock position (**G**, white arrows indicate the PVL).

In our institution, we compared 3D TEE images (EPIQ CVx, Philips Medical Systems, Andover, MA, USA) captured from two patients with severe MAC and a control patient without MAC [32]; in one patient with evidence of MAC, cardiac CT imaging (Brilliance 256-slice iCT, Philips Medical Systems) was also performed and confirmed the presence of severe MAC (Figure 1A,B). While 3D TEE with the routinely used colorized depth and transillumination volume-rendering tools documented a circumferential irregularity of the mitral annulus seen from the surgeon’s view in both the patients with MAC (Figure 1C,D) and the control patient (Figure 2A–C), switching to the MIP volume-rendering tool allowed us to not only identify or exclude (Figure 3A,B) the presence of MAC, but also, when MAC was present, to evaluate its distribution and extent (Figure 3C,D) [32].

The MIP technique may also offer additional value for 3D-guided mitral valve planimetry for the pre-procedural planning of transcatheter edge-to-edge repair procedures and valve sizing for valve-in-mitral annular calcification (valve-in-MAC) transcatheter mitral valve replacement since it allows for the real-time visualization of calcium on the cardiac anatomy.

Besides its role in pre-procedural planning, this tool can also be useful intra-procedurally. Indeed, real-time mitral calcium mapping successfully guided the positioning and deployment of a transcatheter valve-in-MAC procedure at our institution [33]. An MIP map enables the visualization of calcium’s distribution and depth on the cardiac anatomy (Figure 3E) in a more descriptive and live manner compared with CT. During a valve-in-MAC procedure, the MIP tool offered better visualization of the relationship between the valve frame and the mitral annulus, guiding the pre-deployment valve depth and co-axiality adjustment. Post-deployment, unlike the standard colorized depth map, the MIP tool was able to clearly delineate how the valve frame opposed the surrounding calcium (Figure 3F), without significant gaps, allowing for the exclusion of a significant paravalvular leak and post-dilation need, which was confirmed with Color Doppler (Figure 3G) [33]. Color Doppler can be used concomitantly with an MIP; we believe that it should currently be used in combination with color on the standardized 3D colorized-depth map, but a fusion of the two imaging modalities may be possible in the future.

## 5. Conclusions

Echocardiography represents the first imaging modality for MAC assessment; however, compared to cardiac CT, it offers lower specificity with which to distinguish calcium from dense collagen.

The novel 3D TEE MIP tool allows for the identification of MAC’s presence, distribution, and depth on the cardiac anatomy. Therefore, an MIP map represents a promising tool that can be very useful in clinical practice, since information on the presence, distribution, and extent of calcium are critical for the pre-procedural planning and intra-procedural guidance of mitral valve interventions in order to reduce the risk for complications.

## Data Availability

No new data were created or analyzed in this study. Data sharing is not applicable to this article.
